# Determinants of HIV testing uptake among partners of pregnant women in Addis Ababa, Ethiopia: a community-based study

**DOI:** 10.11604/pamj.2021.39.7.27839

**Published:** 2021-05-03

**Authors:** Yanet Taklemariam Gizaw, Moges Muluneh Boke, Alehegn Bishaw Geremew

**Affiliations:** 1 Marie Stope International, Addis Ababa, Ethiopia,; 2 Department of Reproductive Health, Institute of Public Health, College of Medicine and Health Sciences, University of Gondar, Gondar, Ethiopia

**Keywords:** Male partner, pregnant women, HIV test

## Abstract

**Introduction:**

couples HIV testing and counseling is an important intervention to make an informed decision on reproductive health, to adopt preventive behaviors, support each other, and prevent mother-to-child HIV transmissions. Despite the importance of partners of pregnant women HIV testing uptake, there is limited study in Ethiopia. Hence, this study aimed to assess the proportion of HIV testing uptake and its determinants among partners of pregnant women.

**Methods:**

a community-based cross-sectional study was conducted from January to February 2020 in Addis Ababa. A multistage cluster sampling technique was used to recruit 812 partners of pregnant women. A pre-tested and structured questionnaire was used to collect the data. Binary logistic regression analysis was performed to identify the determinants of HIV testing uptake among partners of pregnant women. Adjusted odds ratio with 95% confidence interval was used to declare statistical association and the direction of the association between the dependent variable and independent variables.

**Results:**

overall, a total of 63.7% (95% CI: 60-67%) of partners of pregnant women were tested for HIV/AIDS. Knowledge on mother to child transmission of HIV (AOR=2.0, 95% CI: 1.37-3.06), previous history of couple HIV testing and counseling (AOR=3.8, 95% CI: 2.49-5.85), discussion with spouse (AOR= 6.6, 95% CI: 4.44-9.91), and having information about discordant HIV test result (AOR =2.3, 95% CI: 1.48-4.14) were significantly associated with partners of pregnant women HIV test uptake.

**Conclusion:**

HIV testing uptake among partners of pregnant women was low. To increase the uptake of HIV testing, program designers and implementors should work on knowledge of the spouse´s on mother to child transition of HIV, to have more discussion between couples, and consider and strengthen activities that increase couple HIV testing and counseling at the community level before pregnancy.

## Introduction

Globally, an estimated 36.9 million people were living with HIV in 2017 [1]. Each year one million HIV-related death and 1.8 new cases occur in the world. Sub-Saharan Africa (SSA) accounted for 76% of the total new HIV infections and 75% of the total HIV/AIDS-related deaths [2]. HIV epidemic has remained a public health problem in the sub-Saharan Africa region. To combat the epidemic, stakeholders had adopted 90-90-90 targets or HIV testing goals that call for 90% of all people living with HIV to know their status by 2020 [3]. The global HIV testing magnitude has been substantially increased in the past decade, between 2005 and 2015; the proportion of people living with HIV aware of their status shifted from 12% to 60% [4]. However, HIV testing is poor in the Middle East and North Africa, in which 50% and 48% of people know their HIV status, respectively [5]. A significant number of HIV-positive men continued without a diagnosis in sub-Saharan Africa regions. In sub-Saharan Africa, men living with HIV were less aware of their HIV status than women [6]. Due to this reason, men are less likely to access HIV treatment and care and more likely to die due to AIDS-related illnesses than women [3].

To achieve HIV zero generation targets and to reduce the risk of mother-to-child HIV transmission significantly, involving male partners in antenatal voluntary HIV counseling and testing (VCT) is the best strategy. Offering VCT services to men at the antenatal clinic with their couple and individual counseling is an essential and acceptable strategy for increasing male involvement in the prevention mother to child transmission (PMTCT) and promoting male HIV testing [7]. Non-clinical setting; community-based testing, self-testing, workplace, and home-based testing are all important approaches that make HIV-positive men aware of their status [4,6]. Despite the alternative approach available, men partner HIV test uptake was stagnant in developing countries [8]. In Ethiopia, a few partners of pregnant women's received HIV testing; for this low uptake of HIV testing and counseling, there are reasons reported by the researchers that the studies have been focused on institution setting and women respondents instead of the male partner [9,10]. The information obtained from women about men's perspective may not bring accurate and reliable evidence to decision-makers and program designers. Therefore, this study aimed to assess HIV testing and counseling uptake and its determinant among partners of pregnant women in Addis Ababa, Ethiopia.

## Methods

**Study design and settings**: a community-based cross-sectional study was conducted in Addis Ababa, Ethiopia from January to February 2020. Addis Ababa city has an estimated density of 5,535.8 people per square kilometer. Based on the 2007 census from Ethiopia´s central statistical agency, Addis Ababa city has an estimated total population of 3.5 million as projected for the year 2014 [11]. The city has ten sub-cities and 116 Woredas. There are 13 public and three private hospitals. Moreover, there are 100 public health centers and around 700 private clinics, out of which 75 are higher clinics. All partners of pregnant women found in Addis Ababa were the source of population, while partners of pregnant women living in the selected sub-city of Addis Ababa during the data collection period were considered as the study population.

**Sample size determination and sampling procedures**: a single proportion population formula was used to determine the sample size. The following assumptions were considered for sample size calculation; 40% proportion of male partner tested in Gondar town [12], 95% level of confidence, 5% margin of error, design effect of 2, and 10% of non-response rate, finally which yields 812. The sample size for determinants was calculated using epi info version 7 software, however, the calculated sample size was found less than the sample size calculated for the first objective. A multistage cluster sampling technique was used. From the total ten sub-cities, three sub-cities were selected by lottery method. Five woredas from each selected sub-city were selected randomly. The annual number of pregnant women from the selected woreda was reviewed. Then the proportional allocation of a sample size to the number of partners of pregnant women was done. Finally, the required number of study participants recruited from each selected woreda until the required sample size was recruited.

### Operational definitions

**Partner HIV testing uptake**: when husbands or partners of pregnant women attended both HIV counseling and testing (HCT) during ANC visit for PMTCT service.

**Knowledge on mother-to-child transmission of HIV (MTCT)**: if respondents knew at least two routes of mother-to-child HIV transmission from three routes of transmission [12], was considered as “good knowledge”.

**Knowledge about PMTCT**: if respondents knew at least two ways of PMTCT from three ways of PMTCT [12], was considered as “good knowledge”.

**Proxy testing**: male partner is assuming to have the same HIV status as that of his partner´s HIV test result.

**Data collection procedures**: an interview administered structured questionnaire developed from published articles was used to collect data [9,12,13]. The questionnaire was initially prepared in English and later translated into the local Amharic language and back to English to keep the consistency. The questionnaire consisted of five sections: socio-demographic information, knowledge, family communication on HIV/AIDS, and counseling and testing experience for HIV. Data were collected while the study participants were at the home or nearby home by the data collectors reached each household where a pregnant woman was available. Re-visited was done for those respondents who were not available at the time of data collection due to different reasons. Fifteen health extension workers and three BSc holder nurses were involved in data collection and supervision, respectively.

**Data quality assurance**: to ensure the data quality, a two-day training was given for data collectors and supervisors on the objective, relevance, and benefits of the study, confidentiality of information, respondent´s right, informed consent, and interview technique before the actual day of data collection. A pre-test was conducted on 41 partners of pregnant women in Yeka sub-city Woreda 06, and some minor modifications were done after the pre-test. The collected data were reviewed and checked for completeness and consistency by the investigators and supervisors daily at the spot during the data collection time.

**Data processing and analysis**: the data were initially entered into Epi-Data version 4.6 and exported into SPSS software version 25.0 for analysis. Before the analysis, the data were coded and cleaned. The missed and data error was cleaned by referring to the filled questionnaire. Descriptive analysis was performed to describe the frequency, percentage, and mean of study participants' socio-demographic characteristics. A binary logistic regression model was fitted to analyze the relationship between the dependent and independent variables. If partners of pregnant women attended both HIV counseling and testing during ANC visit for PMTCT service was considered HIV tested. All variables with a P-value of <0.2 in the bivariable analysis were entered into multiple logistic regression models to identify factors independently associated with HIV testing uptake among partners of pregnant women. The statistical significance of independent variables was determined using odds ratios (OR) with 95% CI.

**Ethical approval and consent for participation**: the ethical approval was obtained from the Institute of Public Health, College of Medicine and Health Science, University of Gondar. A permission letter was secured from the Addis Ababa health bureau. The purpose, benefits, right to refuse anytime from the study were explained for each study participant. Written consent was taken from each study participant before the actual data collection. To keep confidentiality, any personal identification of the study participants was not recorded on the questionnaire.

## Results

**Socio-demographic characteristics of the respondents**: a total of 798 study participants were interviewed, giving a response rate of 98.2%. The mean age of the study participants was 37.5 years, with a standard deviation of ±7.1 years. Approximately half (49.7%) of the study participants were aged between 31-40 years, and almost all (94.5%) study participants had formal education. Slightly more than half (50.8%) of the study participants have lived together in marriage for 1-5 years. More than half (51.3%) of study participants were employed in private work and had an average minimum monthly income of 5,000 Ethiopian Birr (Table 1).

**Table 1:** socio-demographic characteristics of partners of pregnant women in Addis Ababa, Ethiopia, 2020

Variables (Addis Ababa Ethiopia, 2020 (n=798))	Frequency	Percentage (%)
**Age**		
<20 Years	2	0.3
21-30 Years	143	17.9
31-40 Years	397	49.7
41-50 Years	227	28.4
>51 Years	29	3.6
**Duration of marriage in a year**		
<1	99	12.4
1-5	405	50.8
5-10	195	24.4
>10	52	6.5
**Educational status**		
No formal education	44	5.5
Primary school (1-8)	139	17.4
Secondary school (9-12)	280	35.1
College and above	335	42.0
**Occupation**		
Governmental employee	247	31.0
Private	409	51.3
NGO	83	10.4
Daily labor	59	7.4
**Personal average monthly income**		
<1000 ETB	13	1.6
1,100-4,999 ETB	380	47.6
5,000-9,999 ETB	347	43.5
>10,000 ETB	58	7.3
**Age of women**		
<20 Years	14	1.8
21-30 Years	376	47.1
31-40 Years	368	46.1
>41 Years	40	5.0
**Educational status of women**		
No formal education	90	11.3
Primary school (1-8)	198	24.8
Secondary school (9-12)	216	27.1
College and above	294	36.8

**Partners of pregnant women knowledge on HIV transmission and prevention**: of the total study participants, 63.9% were had good knowledge of mother-to-child HIV transmission. The study participant's knowledge regarding prevention mother to child HIV transmission, 38.3% of partners of pregnant women were had good knowledge of PMTCT. Approximately three-fourths (71.2%) of partners of pregnant women knew antiretroviral therapy provision during pregnancy, child labour and delivery, and breastfeeding, 43.6% knew provision of ARTs for the newborn. The majority (85%) of partners of pregnant women knew the availability of HIV counseling and testing service during ANC visits. Three fourth of partners of pregnant women were had information about HIV test discordant results and a prior history of HIV testing and counseling. More than half (60.4%) of study participants were discussed HIV/AIDS and antenatal care (Table 2).

**Table 2:** knowledge on HIV/AIDS transmission and prevention among partners of pregnant women in Addis Ababa, Ethiopia, 2020

Variables	YES	NO
	Frequency (%)	Frequency (%)
**Knowledge of HIV transmission**		
Unprotected sexual intercourse	745 (93.4)	53 (6.6)
Sharing of infected sharp material	695 (87.1)	103 (12.9)
Mother-To-Child transmission	594 (74.4)	204 (25.6)
**Knowledge of mother to the child transmission**		
During pregnancy	613 (76.8)	185 (23.2)
During labour and delivery	544 (68.2)	254 (31.8)
During breastfeeding	532 (66.7)	266 (33.3)
**Knowledge of prevention mother to child transmission**		
Provision ART drugs	568 (71.2)	230 (28.8)
Cesarean section delivery	285 (35.7)	513 (64.3)
Provision ART to the newborn	348 (43.6)	450 (56.4)

**Figure 1:**
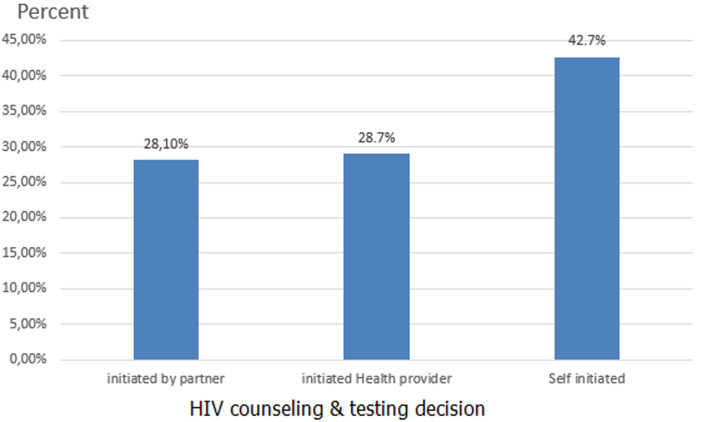
HIV counseling and testing decision made by partners of pregnant women in Addis Ababa, Ethiopia, 2020

**Figure 2:**
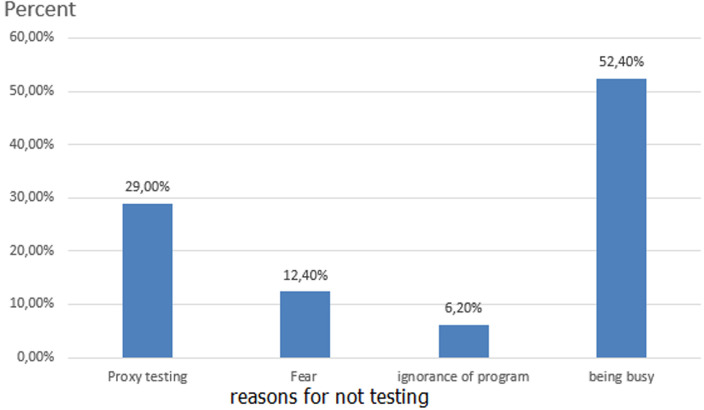
the reasons mentioned for not testing HIV among partners of pregnant woman in Addis Ababa, Ethiopia, 2020

**The proportion of HIV test uptake among partners of pregnant women**: in this study, 63.7% (95% CI: 60-67) of partners of pregnant women were received HIV testing and counseling service with their wives during ANC visits. Of those who received HIV testing, 42.7% of them were decided by themselves, and 28.7% of partners were tested by invitation of health care provider (Figure 1). The main reasons mentioned by the participants for not receiving HIV testing and counseling for partners were fear of test results (12.4%), proxy testing (29%), and being busy (52.4%) (Figure 2).

**Factors associated with HIV testing uptake of partners of pregnant women**: in the bivariable analysis variables such as; educational status, age, employment status, knowledge on HIV transmission, prior history of HIV testing and counseling, couple´s discussion about HCT and ANC services with their spouse, having information about discordant HIV test results were a p-value of less than 0.2 (Table 3). In the multivariable analysis; knowledge about MTCT, prior history of HIV testing and counseling, discussion about HCT and ANC services with a spouse, having information about discordant HIV test results were significantly associated with partner HIV testing uptake. Male partners who knew the route of HIV transmission from mother to child were 2.0 times more likely to receive HIV testing than their counterparts (AOR=2.0, 95% CI: 1.37-3.06). The odds of HIV testing uptake were 3.8 times higher among male partners who had a prior history of HIV counseling and testing than their counterparts (AOR=3.8, 95% CI: 2.49-5.85). Couples who discussed HCT and ANC service were 6.6 times more likely received HIV testing than those who did not discuss it (AOR= 6.6, 95% CI: 4.44-9.91). Male partners who had information about discordant HIV test results were 2.3 times more likely received HIV testing compared to those who did not have the information (AOR =2.3, 95%CI:1.48, 4.14) (Table 4).

**Table 3:** bivariate analysis of factors associated with HIV test uptake of partners of pregnant women in Addis Ababa, Ethiopia, 2020

Variables	HIV tested		Odds Ratio(95% CI)
	
	Yes	No	
**Age**			
20-30 Years	88	57	2.5(0.95-6.47)
31-40 Years	255	142	2.1(0.85-5.36)
41-50 Years	142	85	2.3(0.98-5.86)
>50 Years	23	6	1
**Education status**			
No formal education	14	30	1
Primary school (1-8)	62	77	1.7(.8-3.5)
Secondary school (9-12)	168	112	3.2(1.6-6.3)
College and above	264	71	7.9(4.0-15.8)
**Occupation**			
Governmental employee	195	52	7.3(3.9-13.5)
Private	243	167	2.8(1.5-5.0)
NGO	50	33	3.1(1.5-6.2)
Daily labor	20	38	1
**Knowledge of modes of HIV transmission**			
Good	457	225	2.5(1.7-3.8)
Poor	51	65	1
**Knowledge on mother to child HIV transmission**			
Good	380	130	3.6(2.6-4.9)
Poor	128	160	1
**Awareness of the presence of HCT during ANC visit**			
Yes	472	206	5.3(3.5-8.1)
No	36	84	1
**Ever received a couple of HIV counseling testing prior**			
Yes	440	153	5.7(4.1-8.1)
No	68	137	1
**Discussed HCT and ANC services with their spouse**			
Yes	404	78	10.5(7.5-14.7)
No	104	212	1
**Heard about discordant HIV test result**			
Yes	439	160	5.1(3.6-7.2)
No	69	130	1

**Table 4:** multivariable logistic regression of factors associated with HIV test uptake of partners of pregnant women in Addis Ababa, Ethiopia, 2020

Variables	Partner HIV tested		Adjusted Odds Ratio (95% CI)
	Yes	No	
**Education status**			
No formal education	14	30	1
Primary school (1-8)	62	77	0.8(0.34-2.11)
Secondary school (9-12)	168	112	0.5(0.21-1.25)
College and above	264	71	0.3(0.12-0.79)
**Occupation**			
Governmental employee	195	52	0.4(0.19, 1.06)
Private	243	167	0.8(0.36, 1.06)
NGO	50	33	0.7(0.28, 1.82)
Daily labor	20	38	1
**Knowledge of modes of HIV transmission**			
Good	457	225	0.68(0.38, 1.22)
Poor	51	65	1
**Knowledge on mother to child HIV transmission**			
Good	380	130	2.0(1.37, 3.06) *
Poor	128	160	1
**Awareness of the presence of HCT during ANC visit**			
Yes	472	206	0.6(0.37, 1.08)
No	36	84	1
**Ever received a couple of HIV counseling testing prior**			
Yes	440	153	3.8(2.49, 5.85) *
No	68	137	1
**Discussed HCT and ANC services with their spouse**			
Yes	404	78	6.6(4.44, 9.91) *
No	104	212	1
**Heard about discordant HIV test result**			
Yes	439	160	2.3(1.48, 4.14)*
No	69	130	1

Note * statistically significant at P< 0.05

## Discussion

In this study, about 63.7% (95% CI: 60%-67%) of partners of pregnant women were tested for HIV when their wives received ANC service during the most recent pregnancy period. This finding is higher than the studies´ findings in Cameroon 58.3% [14], Goba 22.7% [9], and Gondar town 40.1% [13], India Georgia 37.5% [15], Dominican Republic pooled estimate 36.1% [16], and South Africa 32% [17]. The possible reasons for this discrepancy might be the availability of infrastructures like an easy way of access for transportation to reach health facilities in our study area than Goba and Gondar. In contrast, the current study's finding is lower than the studies conducted in Rwanda 81% [18] and Uganda 90% [19]. The reason for this discrepancy might be the difference in socio-economic status and health services availability. The study setting variation could be also the other main reason in which the prior studies were conducted at facility level that resulted higher results. In this study, spouses who had good knowledge of MTCT were more likely to be tested for HIV. This evidence is supported by studies obtained from Goba [9] and Gondar [12].

Prior experience of HIV testing and counseling was increased the HIV testing uptake among partners of pregnant women. This evidence was in agreement with studies finding reported from Mekele, Ethiopia [9], and Midlands Province, Zimbabwe [11]. This suggests that promoting or enhancing couple HIV testing and counseling activities like volunteer HIV testing and counseling and others before pregnancy at the community level might enhance partner involvement in HIV testing during pregnancy. Partners' discussion on ANC and HCT issues have significantly increased HIV testing uptake. This finding is in line with that of a study done in Midlands Province, Zimbabwe [14]; This resulted in the couple´s discussion on the essential of HIV testing was more likely accepted for having HIV testing and stick to the PMTCT treatment. This evidence also suggests that improved communication between couples regarding HIV is an important determinant in increasing the number of men accompanying their spouses to antenatal clinics and accessing HIV counseling and testing services [19,20]. This study also revealed the male partners who had an awareness of discordant HIV test results were more likely to have HIV testing during their wives´ ANC follow-up. This finding was supported by a study obtained from Rwanda and Zambia [21]. Some limitations are acknowledged for our study. All responses in this study were self-reported information, and due to this reason, outcome interest might be subjected to social desirability bias.

## Conclusion

In this study, we found that a low proportion of partners of pregnant women were tested for HIV during their wife's current pregnancy. Knowledge on HIV transmission from mother to child, prior experience of HIV testing and counseling, discussion about HCT and ANC service with spouses, and having information on HIV test discordant result was identified as determinants to HIV test uptake of partners of pregnant women. Therefore, education programs need to pay attention to the discordant HIV test result and HIV mother-to-child transmission. Program designers need to consider and strengthen activities that increase couple HIV counseling and testing at the community level before pregnancy, and couple communication on HIV/AIDS-related issues needs to encourage.

### What is known about this topic

- Couples HIV testing and counseling service is important to adopt HIV preventive behaviors and prevent mother-to-child transmission of HIV;
- Previous studies focused on institution setting and women respondents instead of male partners respondents, the proportion of HIV test uptake of partners of pregnant women is low.

### What this study adds

- The study added that partners of pregnant women HIV test uptake in antenatal care visits was lower as compared to other regions and HIV free generation targets;
- Having information about discordant HIV test results and knowledge mother to child HIV transmission, couple discussion, and prior couple HIV testing and counseling experience are determinants of HIV test uptake among partners of pregnant women.
